# PREDIG: Web application to model and predict the enzymatic saccharification of plant cell wall

**DOI:** 10.1016/j.csbj.2023.09.026

**Published:** 2023-09-29

**Authors:** Partho Sakha De, Torben Glass, Merle Stein, Thomas Spitzlei, Adélaïde Raguin

**Affiliations:** a Institute for Computational Cell Biology, Computer Science department, Heinrich Heine University, Düsseldorf, 40225, Germany; b Bioeconomy Science Center (BioSC), c/o Forschungszentrum Jülich, Jülich, 52425, Germany; c Cluster of Excellence on Plant Sciences (CEPLAS), Heinrich Heine University, Düsseldorf, 40225, Germany

**Keywords:** Plant biomass, Lignocellulose, Bio-refinery, Enzymatic saccharification, Web application, Web interface, Biophysical model, Stochastic simulations

## Abstract

Enzymatic digestion of lignocellulosic plant biomass is a key step in bio-refinery approaches for the production of biofuels and other valuable chemicals. However, the recalcitrance of this material in conjunction with its variability and heterogeneity strongly hampers the economic viability and profitability of biofuel production. To complement both academic and industrial experimental research in the field, we designed an advanced web application that encapsulates our in-house developed complex biophysical model of enzymatic plant cell wall degradation. PREDIG (https://predig.cs.hhu.de/) is a user-friendly, free, and fully open-source web application that allows the user to perform *in silico* experiments. Specifically, it uses a Gillespie algorithm to run stochastic simulations of the enzymatic saccharification of a lignocellulose microfibril, at the mesoscale, in three dimensions. Such simulations can for instance be used to test the action of distinct enzyme cocktails on the substrate. Additionally, PREDIG can fit the model parameters to uploaded experimental time-course data, thereby returning values that are intrinsically difficult to measure experimentally. This gives the user the possibility to learn which factors quantitatively explain the recalcitrance to saccharification of their specific biomass material.

## 1 Introduction

The transition of our current energy landscape from being fossil fuel based to more renewable sources is challenging and requires a combination of measures to phase out our fossil fuel dependence. One of the approaches is the increased production of conventional and advanced biofuels for use in the transport sector. According to the European regulation, advanced biofuels are defined as liquid or gaseous fuels generally produced from cellulosic and lignocellulosic materials, such as agricultural and forestry residues, wastes, energy crops, or aquatic biomass, that have a much lower carbon footprint as compared to fossil fuels. Moreover, such biofuels can be utilised in the existing engines and infrastructures, which is a massive advantage for an alternative energy source [1]. However, recent assessments state that current production levels of advanced biofuels are not satisfactory and a major upheaval is required in this area in the coming years [2].

Lignocellulosic biomass or dry plant matter (e.g. corn stover, sugarcane bagasse, straw, cassava stems, etc.) from agricultural waste is a major feedstock for bio-refinery approaches, not only for the production of biofuels, but also for that of a number of valuable chemicals. In order to convert this biomass into a usable energy source, the major constituent bio-polymers, i.e. cellulose and hemicellulose, are first enzymatically hydrolysed into their respective simple sugar monomers, which are then fermented, to produce bio-ethanol. The most common concept is the initial physical diminution step, during which larger pieces of the biomass are broken down into smaller, more easily managed, fragments e.g. wood chipping. This is usually followed by a pre-treatment step where the biomass is subjected to various aggressive physical and chemical conditions, with the objective to increase the monomers' conversion yield during the downstream saccharification step. However, the heterogeneity and variability of this biomass, originating from a variety of sources, combined with its inherent recalcitrance to enzymatic digestion, is a key hurdle to be overcome for the economic viability of advanced biofuel production. The complexity of this challenge calls for a multidisciplinary approach that incorporates advanced computational techniques alongside existing biotechnological efforts.

Biofuel production from various feedstocks is largely assisted at the system-level by software tools that are either commercialised by companies, or developed by researchers, for instance, to assess market strategies and supply chains [3], [4], [5]. In the present article, we instead focus on the biophysical properties and constraints of lignocellulosic material's degradation at the molecular level. Thus, we are undoubtedly interested in addressing questions that cannot be tackled by the aforementioned methods. Lignocellulose structure has been simulated using theoretical approaches ranging over multiple scales, the classification of which has been well described in the comprehensive review by Ciesielski et al. [6]. At the lowest scale, these models can simulate processes as diverse as: pyrolysis [7], [8], the detailed structure and properties of lignocellulosic biomass [9], enzyme mechanisms [10], [11], and the effects of lignin binding on cellulose and cellulase enzymes [12]. The typically used methods for such detailed models are Density Functional Theory (DFT) and Molecular Dynamics (MD). These are all-atom models based on quantum or molecular mechanics. However, despite remarkable improvements in computational power and high performance computer facilities, all-atom methods are hardly able to depict bio-polymers at the nanoscale, and those can only be simulated for very short amounts of real-time. To overcome these limitations, some coarse-grained MD methods have been developed using beads or pseudo-atoms as elementary units [13]. For instance, Kumar and Murthy [14] studied the enzymatic digestion of a cellulose bundle under the action of endoglucanase (EG), cellobiohydrolase (CBH), and *β*-glucosidase (BGL), by considering glucose molecules as the minimal substrate building blocks. They combined Monte Carlo simulations and experimental methods to investigate the effect of enzyme crowding. In their model, they also accounted for cellulose crystallinity, but without assessing its impact on the saccharification dynamics. In 2017, these authors published an upgraded version of their model, which showed improved comparability with experimental saccharification time-course data. Nonetheless, the discrepancy between experimental and theoretical results remained noticeable, and the modelled substrate consisted of cellulose only, accounting for neither hemicellulose nor lignin. Using instead an Ordinary Differential Equation based approach to implement a mechanistic and kinetic model, Griggs et al. [15], [16] simulated the action of a cellulase enzyme cocktail on a purely cellulosic substrate. This model highlighted the enzymes' synergism and showed a good agreement with experimental cellulose chain length distributions from literature. Later on, the model's extension included both crystalline and amorphous regions, allowing to reach semi-quantitative agreement with both experimental saccharification time-course data and cellulose chain length distributions [17]. However, one could remark that significant simplifications subsisted in the modelling approach, as the substrate was still purely made of cellulose. Besides, using a three-dimensional agent-based model, Vetharaniam et al. [18] simulated the cell wall digestion of perennial ryegrass mesophyll cells by Cel51A, Cel9D, and endoxylanase 1 enzymes. This model, that accounted for hemicellulose sugars, as well as the crystallinity of cellulose, allowed to discuss enzymatic synergism. Nevertheless, the authors reported neither quantitative comparison between simulation results and experimental data, nor the impact of crystallinity on the model's dynamics. Since they exclusively focussed on the primary cell wall, they also discarded lignin from the substrate. Likewise, using agent-based modelling approaches, Asztalos et al. [19] investigated the synergistic action of multiple cellulases. Their detailed model included several steps, such as inter-chain hydrogen bond breaking, hydrolysis of glycosidic bonds, and both adsorption and desorption of the cellulases on the substrate. However, this model limited itself to surface reactions on a simplified two-dimensional grid of glucose, while in addition, hemicellulose and lignin were not investigated. Hence, it is evident that a comprehensive model that accurately represents the lignocellulose substrate properties, and closely compares enzymatic saccharification dynamics to experimental data, is currently lacking. In this backdrop, our biophysical model addresses many of the limitations found in previous modelling approaches [20].

In the present article, we give full and easy access to this complex biophysical model [20], by encapsulating it into a free, open-source, and user-friendly web application (PREDIG), that allows the user to run simulations on our local server. Thereby, the web application PREDIG contributes to the field by empowering researchers with a software tool to simulate the plant biomass enzymatic saccharification process *in silico*, in an unprecedented manner. Our in-house developed biophysical model employs a Gillespie algorithm to conduct stochastic simulations of the enzymatic digestion of a single lignocellulosic microfibril, at the mesoscale, in three dimensions. The model represents not only the three-dimensional structure and the molecular composition of the microfibril, but also the distinct mechanisms, substrate specificity, and kinetics of the enzymes present in typical saccharification cocktails. It also takes into account known sources of lignocellulose recalcitrance to digestion, such as: i) the structural blocking by lignin and hemicellulose of the cellulases' access to their substrate, ii) non-productive adsorption of enzymes onto lignin, iii) crystallinity of cellulose and hemicellulose, iv) end-product inhibition of cellulases by cellobiose and glucose, v) steric hindrance due to the finite size of enzymes, and vi) ‘defects’ in the crystalline structure of cellulose and hemicellulose, arising due to pre-treatments. All these features are quantified, and can be tuned *via* the model's parameters. Noticeably, this biophysical model has already proven its efficacy to decipher experimental saccharification time-course data, and rationalise complex results [20]. By fitting the model's parameter values to experimental saccharification time-course data from the literature [21], we showed that changes in the molecular composition of the substrate after pre-treatments of increasing intensity, are not able to explain the drastic increase in saccharification yield, to which they only marginally contribute, unlike previously deduced from the experiments only. Instead, our model showed that changes in the substrate crystallinity are able to explain such variations in saccharification yield [20].

Thus, owing to the underlying biophysical model's flexibility and ability to quantitatively compare to experimental data, the scope of questions that can be answered by PREDIG is vast. For instance, PREDIG allows the user to test different enzyme cocktails on a specific substrate, towards identifying the most suitable one to increase sugar conversion yield, or for studying enzymatic synergism. Additionally, thanks to the fitting procedure, PREDIG can be used to infer values of the parameters that are difficult to measure experimentally, such as: enzyme kinetic rates, inhibition by end-products, non-productive adsorption of enzymes onto lignin, and crystallinity of cellulose and hemicellulose. Importantly, the software returns quantitative results whose quality depends on the experimental data provided for fitting, both in terms of their quality and quantity.

## 2 Materials and methods

All metadata of the web application and the biophysical model are detailed in Table 1. In the following section, the first two subsections describe the architecture and functionalities of PREDIG. The third one focusses specifically on briefly presenting the biophysical model that simulates the enzymatic saccharification of the lignocellulose microfibril, and the associated fitting algorithm.

**Table 1:** Codes metadata.

Current code version	v1.0.0
Link to the repositories used for this code version	Web application: https://github.com/Iamsecret/PREDIG-Web-Application; Biophysical model & fitting algorithm: https://github.com/psde-777/PREDIG_biophysical_model_N_fitting_algorithm

Legal Code Licenses	Web application: AGPL-3.0; Biophysical model & fitting algorithm: GPL-3.0; Results produced by our codes (figures, videos, raw data...): CC BY 4.0

Copyright	Web application: Copyright notice; Biophysical model & fitting algorithm: Copyright notice

Software code languages, tools, and services used	Web application: Python, ReactJs, NodeJs, MariaDB, Docker; Biophysical model & fitting algorithm: C++, Python, Bash

Compilation requirements, operating environments & dependencies	Web application: Docker; Biophysical model & fitting algorithm: Linux OS/VM, C++, Python 3 (libraries: matplotlib, scipy, pyfiglet, numpy, glob, os, re, tqdm, imageio, sys, random, math, PIL) and ffmpeg.

Available link to documentation	Web application: README; Biophysical model & fitting algorithm: README

### 2.1 Software architecture

**Fig. 1:**
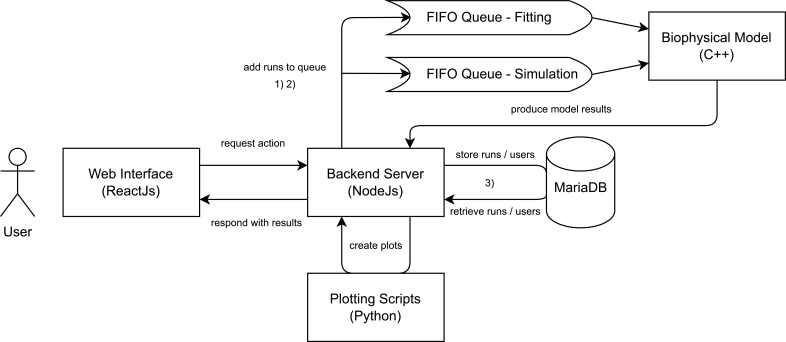
Schematic representation of PREDIG's software architecture. Actions that can be requested by the user are numbered as: 1) simulation, 2) fitting, and 3) retrieving run results.

The architecture of the web application PREDIG, as shown in Fig. 1, is divided into a frontend written in ReactJs and a backend written in NodeJs, that is itself connected to a MariaDB database. The frontend is reachable at https://predig.cs.hhu.de/ and is the only part interfacing with the user. There, the user can choose which action they want to perform among: 1) simulation, 2) fitting, and 3) retrieving run results. Details about them are presented in the Section 2.2. The backend service coordinates and performs these actions, as well as stores the results of the simulations and the fitting procedure. Using Python scripts, it also creates the associated plots, that are displayed to the user in the RUNS tab of the web interface. Because of the different computing times and resources (i.e. availability of CPU threads) necessary for running a simulation *versus* a fitting procedure, each of these actions are placed in distinct queue data structures, that are managed independently, and for which dedicated specific computing resources have been allocated on our server. For each queue, the backend handles requests for actions in a first come first serve manner, by checking at regular time intervals if enough resources are available to perform a new action. Considering the limited size of the data produced by either the simulations or the fitting procedure, the action of retrieving these data does not require being managed by a queue. Finally, the backend service sends out emails to the user, to both ask for the confirmation of the action they want to perform, and to notify them when a requested action is finished.

### 2.2 Software functionalities

Using PREDIG's web interface, the user can perform one of three actions. They can: i) simulate the enzymatic digestion of a substrate microfibril with the model, ii) fit the model's parameter values to experimental saccharification time-course data, and iii) search for finished runs submitted by either themselves or other users. Each of these actions are interfaced by a distinct tab. They are guided by extensive instructions, including literature references.

**Fig. 2:**
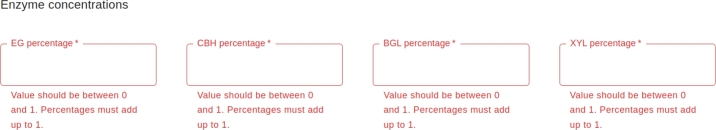
Validation step checking that all parameters belonging to the same distribution sum up to one, for the case of the enzyme concentrations. The error messages also display the value ranges for the percentages of EG, CBH, BGL, and XYL enzymes that compose the saccharification cocktail.

**Simulation of the saccharification process.** On the SIMULATION tab of the web interface, the user must set both the kinetic parameters and the initial configuration parameters. Full details about these parameters (e.g. their meaning and value range) are available in the Appendix B. On the interface, we provide pre-filled values for all parameters, which can always be accessed again by pressing the RESET TO DEFAULT VALUES button in each box. This allows the user to readily play with the model, and to reproduce the case of high pre-treatment severity in the illustrative example described in the Section 3. Parameters can also be entered by uploading a json formatted parameter file, for instance obtained from a previous run, by clicking the button DOWNLOAD PARAMETER FILE on the RUNS tab. For each parameter that the user can vary, specific information on their biophysical meaning and value range is provided when hovering the cursor over them. Additionally, if setting parameter values that do not comply with the provided instructions, the software gives feedback by turning red and displaying an error message. As illustrated on Fig. 2, for parameters that are percentages, this validation step also includes checking that all parameters belonging to the same distribution sum up to one. Such feedback enhances the usability of the software and is a direct benefit of using the web interface.

Some parameters of the biophysical model cannot be varied using PREDIG, for distinct reasons, such as to limit computing time on the hosting server (e.g. the length of the microfibril). For completeness, all parameters of the biophysical model are reported in Appendix B, together with their meaning, value range, and status on the web interface (fixed or variable). For enzyme kinetic parameters, i.e. $k_{\mathrm{cat}}$ and $K_{m}$, we use values reported in the BRENDA database [22] to offer the user with pre-tabulated options from distinct micro-organisms (see Appendix A for details). To start a simulation run, the user must provide a valid email address and click on the START THE SIMULATION button. Here the user also has the option to request a video clip of the simulated three-dimensional saccharification process. They will receive a first email for them to confirm the start of the simulation, and a second one notifying the completion of the run, whose results are accessible by a link. This aims at avoiding spam and ensuring that a run is always linked to an actual email address.

**Fig. 3:**
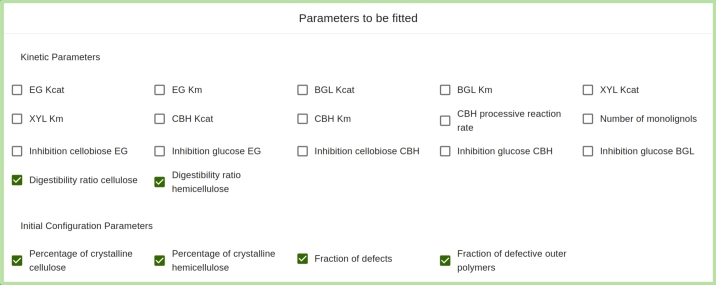
Box for choosing the parameters to be fitted. These can either be part of the kinetic parameters or the initial configuration parameters.

**Fitting model's parameter values to experimental data.** A similar interface is provided for the fitting procedure. Additionally, here (see Fig. 3) the user has to choose which parameters to fit to best reproduce the uploaded experimental data. Note that not all parameters are available to be fitted. Parameters that are not available for fitting or not chosen to be fitted, stay fixed during the fitting procedure.

**Fig. 4:**
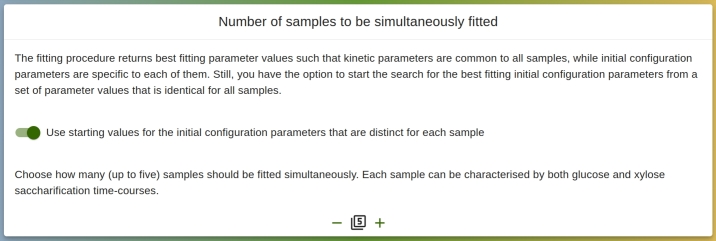
Box for the user to decide how many distinct samples (that may include both glucan and xylan saccharification time-course data) are to be simultaneously reproduced in the fitting procedure.

**Fig. 5:**
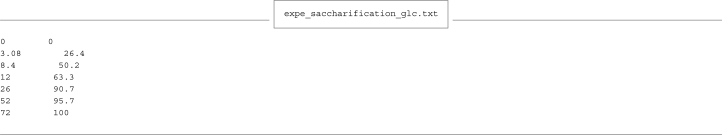
Illustrative example available to be downloaded from the FITTING tab, that shows the format of an experimental time-course data file for glucan conversion of corn stover biomass after high intensity pre-treatment, taken from the work of Bura et al. [21]. The first column contains the time in hours, the second one the percentage of glucan conversion.

**Fig. 6:**
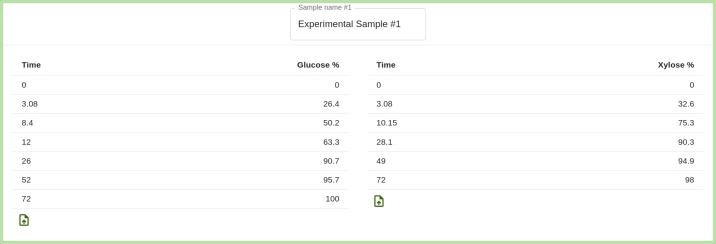
Preview of the saccharification time-course data files after they have been uploaded by the user on the web interface. Here, the name of the sample can also be specified.

The user must upload experimental glucose and/or xylose saccharification time-course data, for up to five different samples, as can be seen in Fig. 4. All saccharification time-course data should come from experiments following the same protocol, which implies that some parameters are common for all samples. Fitting multiple saccharification time-courses simultaneously has the advantage of providing additional constraints to the fitting algorithm, thus improving the validity of the results. As a trade-off, it is more difficult for the fitting procedure to find suitable parameter values accurately fitting all data sets. As illustrated on Fig. 5, the experimental data should be provided in a tab-separated utf-8 encoded text file, with the first column representing the time (in hours) and the second one the percentage of glucan/xylan conversion. Fig. 6 shows how the experimental saccharification time-course data are displayed on the interface after their upload. For the initial configuration parameters, by default, the user has to supply values distinct for each uploaded sample. Alternatively, they can choose to set them all to identical values. In either case, those values will be used as initial starting points for the fitting procedure. Note that, regardless of how the starting values are selected, the set of initial configuration parameters returned by the fitting procedure is unique for each sample.

**Retrieving results.** Navigating the RUNS tab on PREDIG's web interface, the user can retrieve any fitting or simulation result stored on the server. More precisely, the user is presented with the list of all runs performed so far, with the most recent ones at the top. To search for their own results among this wealth of data, the user can either use the links provided per email, after the completion of their runs, or use the email address filter.

**Fig. 7:**
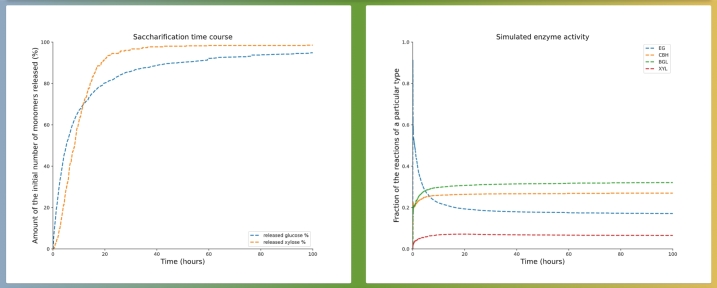
Simulation results for the default parameter values pre-filled on PREDIG's web interface, tab SIMULATION. Those values have been obtained for the best fit of the high pre-treatment severity case described in the illustrative example in Section 3. (left) saccharification time-courses for glucose and xylose, (right) enzyme activity. These results can be found on the RUNS tab of PREDIG's web interface using the run details: - UUID: ffabd05f-ab49-4149-8a76-3a51b5a8ea3d, submitted by: partho.de@hhu.de.

As shown on Fig. 7, simulation results include two plots. On the left, both the glucose and xylose conversions through time are displayed as percentages of the initial total amount of monomers of their respective type. On the right, the relative activity through time is recorded for each enzyme. By clicking DOWNLOAD DATA, the user obtains the zipped simulation directory, which contains all the parameter values in plain text files, as well as the compiled code binary, and the data for both of the plots, allowing further analysis. One should carefully note that these parameter files cannot be directly uploaded back on the interface. Instead, to easily reproduce or modify a previously run simulation (on the SIMULATION tab), the user must retrieve the json formatted parameter files accessible by clicking DOWNLOAD PARAMETER FILE. Noticeably, if selected when submitting the simulation run, the user has the opportunity to also download the video clip (.mp4 format) that shows the full three-dimensional saccharification process, resolved at the monomer level, for the three types of polymers comprised in the substrate, with a 360° view.

**Fig. 8:**
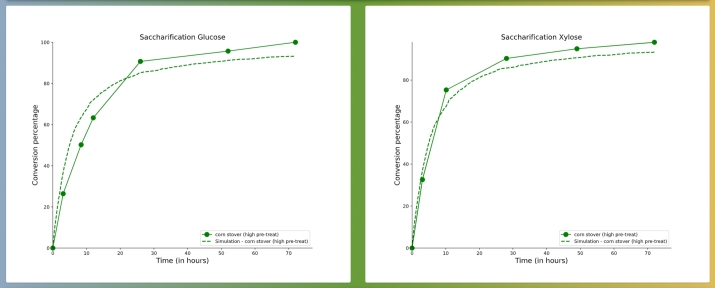
Results obtained for a fitting run, showing fitted saccharification time-courses (dashed lines) superposed to experimental data (points connected *via* solid lines) for (left) glucose and (right) xylose. The experimental data correspond to the high pre-treatment severity case [21] described in the illustrative example in Section 3. These results can be found on the RUNS tab of PREDIG's web interface using the run details: - UUID: 0cfeecfd-57e8-48ff-bc54-644466a24f51, submitted by: partho.de@hhu.de.

As shown on Fig. 8, fitting results include two plots. On the left, the fitted saccharification time-course overlays with the uploaded experimental conversion data, for glucose. On the right, the analogous plot is presented for xylose. The interface also displays, side by side, the starting and the best-fitted parameter values (in bold), for each fitted parameter, thereby highlighting their difference. By clicking DOWNLOAD DATA, the user obtains a zipped directory, which contains the data for both of the plots, the compiled code binary, the fitted parameter values in plain text files, as well as all the Python and shell scripts required for running the fitting algorithm locally. By clicking DOWNLOAD PARAMETER FILE, the user is provided with similar information like in the case of simulation results. In addition, to evaluate the quality of the fits obtained, the coefficient of determination $\left(R^{2}\right)$ is displayed for each fit, i.e. for both glucose and xylose saccharification time-courses, for each sample.

### 2.3 The stochastic biophysical model and the fitting algorithm

**Implementation and dependencies.** The algorithm of the stochastic biophysical model sums up to ca. 9,000 lines of C++ code and is compiled using GCC version 10.2.1 20210110 (Debian 10.2.1-6) on 64-bit Linux. The fitting algorithm employs a few Python scripts, summing to ca. 500 lines, to vary the model input parameters, and to compare the model output to the experimental saccharification time-course data. Additionally, some Bash wrapper scripts are used to: i) manage the distribution of the tasks on the available CPU threads, ii) clean and sort the output files after each simulation run, and iii) save the best-fitted parameter values. Both the simulation code and the fitting algorithm can easily be run locally on Linux based operating systems, provided that the requirements mentioned in the above Table 1 are fulfilled. To run these codes locally on Windows OS, a Docker container or a Linux Virtual Machine are recommended.

**Fig. 9:**
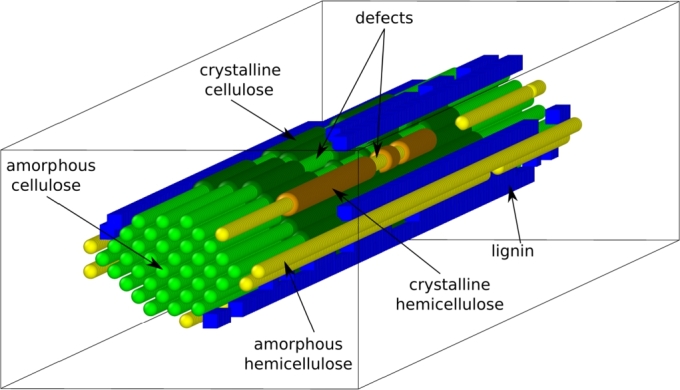
Perspective view of the modelled lignocellulose microfibril substrate, with lignin in blue, hemicellulose in yellow, and cellulose in green. Both hemicellulose and cellulose can be either crystalline (dark colour) or amorphous (light colour), with amorphous regions typically located at the boundaries of the microfibril, or embedded in the crystalline region as ‘defect’ patches. These defects may for instance arise as a result of pre-treatments.

**The stochastic biophysical model.** The stochastic biophysical model employs a Gillespie algorithm to simulate in three dimensions the enzymatic saccharification of a single lignocellulose microfibril. The structure of the substrate is resolved in three dimensions at the monomeric level: glucose for cellulose, xylose for hemicellulose, and monolignols for lignin. At its core, the microfibril comprises a bundle of cellulose polymer chains of the same length, surrounded by layers of hemicellulose and lignin, as seen in Fig. 9. The length of the microfibril is specified in terms of the number of bonds in a cellulose chain (fixed to 200 in the web application). The shape of the microfibril cross-section, as well as the number of cellulose chains, is determined by the plant species used as the material source. The composition of the microfibril can be tuned in terms of the relative fractions of cellulose, hemicellulose, and lignin, that sum up to 1. Similarly, the relative composition of the enzymes present in the degrading cocktail can be adjusted. The model represents the mechanism, substrate specificity, and kinetics of each for the individual cellulases (i.e. EG, CBH, and BGL), unlike for xylanase (XYL) whose kinetics only is considered, and that, equally for each bond. The known factors contributing to the biomass recalcitrance to enzymatic digestion are accounted for in our biophysical model, and the values of the corresponding parameters can be varied. These are: the structural blocking of cellulases by hemicellulose and lignin, the non-productive adsorption of enzymes onto lignin, the crystallinity of both cellulose and hemicellulose, the end-product inhibition of cellulases, the steric hindrance due to the finite size of enzymes, and the ‘defects’ in the crystalline structure of cellulose and hemicellulose arising due to various pre-treatment processes. Further details about the model and its properties can be found in our previous publication [20]. The stochastic biophysical model employs a total of 50 input parameters, that are read from simple text files, named as ‘simulation_parameters.txt' (14 parameters), ‘kinetic_parameters.txt' (18 parameters) and ‘initial_configuration_parameters_*.txt' (18 parameters). For completeness, all parameters of the biophysical model are reported in Appendix B, together with their meaning, value range, and whether they can be varied when using the web interface. Reasons for not letting the user play certain parameter values are quite distinct. They range from ensuring reasonable computing time on our local server, to presenting the user with the most likely saccharification scenarios by limiting options.

**The fitting algorithm.** The in-house developed fitting algorithm finds suitable model parameter values for accurately reproducing experimental saccharification time-course data. It optimises a subset of chosen model parameters $\left\{p_{i}\right\}$ through recursive generations $\left\{G_{j};\;j=1,2,...,N_{gens}\right\}$, each divided into sub-generations $\left\{SG_{j,k};\;j=1,2,...,N_{gens};\;k=1,2,...,N_{subsets}\right\}$. Across sub-generations of a given generation, the parameter values are randomly varied within a specified range $\left[p_{i,j}-\Delta p_{i,j};\;p_{i,j}+\Delta p_{i,j}\right]$, and the variance between the experimental time-course data and the averaged (over multiple runs) simulation curve is recorded for each sub-generation. If the variance decreases ($var(SG_{j-1,k_{min}})>var(SG_{j,k_{min}})$), the sub-generation with the lowest variance ($SG_{j,k_{min}}$) becomes the starting point for the next generation ($G_{j+1}$), and the gradient thereby found in the parameter space is followed for defining the sub-generations of that new generation ($SG_{j+1,k}$). If instead, the variance increases ($var(SG_{j,k_{min}})>var(SG_{j-1,k_{min}})$), the previous generation's $\left(G_{j-1}\right)$ parameter values are used as a starting point again, and random parameter values are assigned in the range $\left[p_{i,j-1}-\Delta p_{i,j-1};\;p_{i,j-1}+\Delta p_{i,j-1}\right]$. After multiple generations, this hybrid procedure mixing random and directed search converges to a local or global optimum. Finally, the $var(SG_{j,k_{min}})$ across all generations are tabulated, and among them, the minimum is selected, thereby defining the best fit. For each simultaneously fitted experimental time-course data, we save in a separate directory, their best fit and the corresponding parameter values.

Running the fitting algorithm on a local computer allows the user to tune its settings, that are all kept fixed in PREDIG, for optimal utilisation of the available computing resources and the time. These settings are, the maximum number of generations $N_{Gens}$, the number of sub-generations $N_{subsets}$, the percentage range (Δ) for varying the model parameter values, and the number of CPU threads to be used. Complete details about the settings of the fitting algorithm are available in Appendix C. Nonetheless, PREDIG has the major advantage that it spares the user the need for both the technical expertise in computing and managing computation power resources, and the access to such computing resources, also considering that the fitting procedure, in particular, is expensive in that regard. For a complete presentation of the stochastic biophysical model and the fitting algorithm, the reader is referred to our previous article [20].

## 3 Results

### 3.1 Illustrative example

The PREDIG web application does not only grant the user the ability to simulate saccharification of lignocellulose through time, but also to fit the model's parameter values to experimental time-course data. Thereby, PREDIG allows a better understanding of the substrate and the various factors contributing to its recalcitrance to enzymatic digestion. For example, in the case of the experiments done by Bura et al. [21] on corn-stover samples, the increase in glucan conversion by the cellulases was attributed by the authors to the reduction of hemicellulose content in the substrate following pre-treatments of increasing severity. However, in our earlier publication [20] releasing the biophysical model and the fitting algorithm (now both encapsulated in PREDIG), the model revealed that the reduction of the substrate crystallinity with increasing pre-treatment severity, played the key role in leading to the glucan conversion increase.

**Fig. 10:**
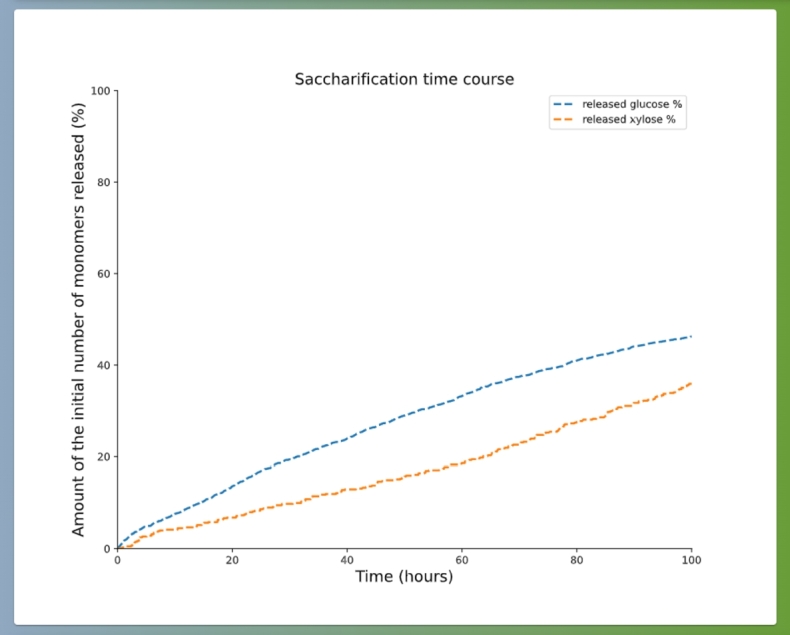
Simulation results for the default parameter values pre-filled on PREDIG's web interface, tab SIMULATION, excepted that the percentages of crystalline cellulose and hemicellulose are both set to 95%. After 72 hours, glucose conversion amounts to about 38% and that of xylose to about 24%. These results can be found on the RUNS tab of PREDIG's web interface using the run details: - UUID: b9c6eb63-23ab-4380-ba76-6efe1c6bd320, submitted by: partho.de@hhu.de.

Here we additionally demonstrate the ability of PREDIG by focussing on fitting the high pre-treatment severity case, from the above described experimental data [21]. To do so, we set the crystallinity to 95%, that we know to be very far away from the expected value, as it results in very low saccharification yield (see Fig. 10 showing the simulation results). However, after only ca. 5 hours, the fitting algorithm was able to accurately reproduce both glucose and xylose conversion time-course curves (see Fig. 8), indeed revealing a very low crystallinity value of ca. 2%. These best-fitted parameter values are now pre-filled as default parameter values on both the SIMULATION and the FITTING tabs of PREDIG's web interface. For completeness, the corresponding experimental data are also provided on the FITTING tab of PREDIG's web interface as “SAMPLE FILE GLC” and “SAMPLE FILE XYL” for the conversion of glucose and xylose, respectively. There, they also serve the purpose of illustrating how such input files must be formatted (see Fig. 5 and detailed explanation in Section 2.2).

### 3.2 Model's predictive capabilities

**Fig. 11:**
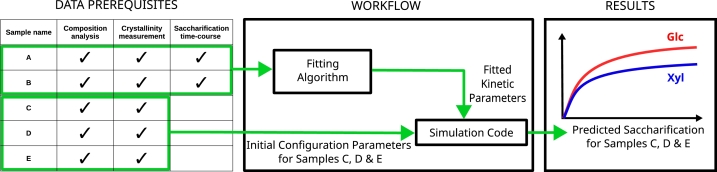
Sketch of the workflow to predict experimental saccharification time-courses. The user first fits time-course data for samples A and B to obtain fitted kinetic parameter values, which they afterwards use, in combination with composition and crystallinity data for samples C, D, and E, to finally predict the saccharification time-courses for the latter ones.

PREDIG can also be used to attempt to predict saccharification time-courses for different samples, measured following the same protocol. The typical workflow for obtaining such a prediction is illustrated by Fig. 11. Here, a user has five samples named as: sample A, B, C, D, and E, for each of which they know the molecular composition and crystallinity parameter values, but the saccharification time-course data for only A and B (see the data prerequisites in Fig. 11, left panel). First, the user fits the model's kinetic parameter values to reproduce the saccharification time-course data for the samples A and B. After collecting the results using DOWNLOAD PARAMETER FILE on the RUNS tab, the user uploads the fitted kinetic parameter values, and enters the molecular composition and crystallinity data on the SIMULATION tab for the samples C, D, and E only (see Fig. 11, middle panel). By running the simulation, the user now obtains a putative prediction for the saccharification time-courses of the samples C, D, and E (see Fig. 11, right panel).

## 4 Conclusion

Enzymatic saccharification of plant biomass, for the extraction of simple sugar monomers and other valuable chemicals, is a central pillar of Bioeconomy, and a precious source of more sustainable and greener energy. Therefore, it is deeply investigated from an experimental and biotechnological point of view, towards achieving economic viability. Yet, the complexity of this system, largely owing to biomass' variability and heterogeneity, is such that unravelling results, and in particular quantifying the impact of specific biophysical features responsible for lignocellulose recalcitrance to saccharification, is challenging. In parallel to experimental research, only a few theoretical models have been developed. Their limitations have been discussed in Section 1, and many of them suffer from a lack of comparability with experimental data.

For the first time, PREDIG fills this critical gap for Biotechnology development. It is designed to significantly change the daily practice of experimentalist researchers by giving them full and easy access to an advanced model of lignocellulose saccharification, that has already proven its ability to compare with experimental data [20]. Among other things, our user-friendly web application alleviates any technical hassle related to computing, such as installing software on a local machine, meeting specific dependency requirements for different operating systems and hardware, and getting access to a high performance computing facility. With PREDIG, the user can readily: i) simulate the enzymatic digestion of a substrate microfibril with the model, ii) fit the model's parameter values to experimental saccharification time-course data, and iii) search for finished runs submitted by either themselves or other users. Importantly, PREDIG does not only permit to answer new targeted questions that are difficult to test experimentally, for instance, determining the most effective enzyme cocktail for any particular type of biomass, or inferring values for specific parameters (e.g. enzyme kinetic rates, inhibition parameters, parameters relating to lignin adsorption on the enzymes etc., for details see the Section 3). It is based on a quantitative approach that also allows to assess our overall understanding of the saccharification process dynamics. Following a typical Systems Biology approach, PREDIG integrates in a complex model features that are experimentally independently measured. Thereby, it contributes to rationalising the larger picture of plant biomass saccharification, drawing new hypotheses, and augmenting the most recent ideas within the domain.

Although no spin-off companies have been launched so far from PREDIG's results, PREDIG is intrinsically meant to rapidly and smoothly spread within the intended user community, comprising researchers from both academia and industry, upon publication and release of the software. To respectively ease and evaluate dissemination, we chose licensing terms compatible with commercial use, and implemented a use-counter on the RUNS tab of the interface. Finally, PREDIG is designed to gather a community of users that openly upload their data, thereby progressively building an unprecedented wealth of time-course saccharification data. This transparent data-sharing will also contribute to changing the daily research practices of the users. By being searchable, preceding runs available in the RUNS tab support the setting of starting parameter values in the fitting procedure. Above all, from these fitting runs fed with experimental data, we will have the possibility to refine the model, and thereby strengthen its predictive power. These future developments of PREDIG will also be boosted by its free and open-source nature, ensuring that it will continue to be improved by the scientific community.

## Funding

The scientific activities of the Bioeconomy Science Center were financially supported by the Ministry of Culture and Science within the framework of the NRW Strategieprojekt BioSC (No. 313/323-400-002 13), that funds the positions of PSD and TG. The position of MS is funded by the 10.13039/501100001659Deutsche Forschungsgemeinschaft (DFG) under Germany's Excellence Strategy EXC 2048/1, Project ID: 390686111. The position of AR is funded by the German federal and state programme Professorinnenprogramm III for female scientists and by the 10.13039/501100001659Deutsche Forschungsgemeinschaft (DFG, German Research Foundation) – Project number: 470067901.

## CRediT authorship contribution statement

**PSD:** supported the development of the web application for all aspects relating to the biophysical model and its associated fitting algorithm, contributed to the web application testing and to designing its legal release terms. Equally with AR, he contributed to writing the almost entire original draft and the revised manuscript, and prepared some of the visualisation for the manuscript. **TG:** programmed and developed the entire web application (including its interface), performed its validation and its curation. He contributed to writing the original draft, to a low extent, and prepared some of the visualisation for the manuscript. **MS:** collected data from the literature and the database BRENDA on enzymes' features. **TS:** DevOps, system and infrastructure administrator, he ensured the deployment of the web application, and provided technical, legal, and security consultancy. **AR:** designed the content and look of the web application, conceptualised the entire project and its methodology. She supervised the work of TG, PSD, and MS, and administrated the whole project. She provided the resources and acquired the funding for her position, as well as that of PSD, TG, and MS. Equally with PSD, she contributed to writing the almost entire original draft and the revised manuscript.

## Declaration of Competing Interest

The authors declare that they have no known competing financial interests or personal relationships that could have appeared to influence the work reported in this paper.

## Figures and Tables

**Table A.2:** Endoglucanase (EC 3.2.1.4) values tabulated on PREDIG's web interface.

Organism	Substrate	*k*_cat_ [1/s]	$K_{m}$ [mM]	experimental conditions	source
*Trichoderma reesei*	4-methylum-belliferyl cellobioside	0.01	0.125	pH=5.0, 45Â°C	BRENDA

*Pyrococcus horikoshii*	p-nitrophenyl cellobiose	0.41	0.95	pH=5-6, 50Â°C	BRENDA

*Rasamsonia emersonii*	4-methylum-belliferyl beta-D-lactoside	0.52	0.33	pH=5.0, 22Â°C	BRENDA

**Table A.3:** Exoglucanase (EC 3.2.1.91) values tabulated on PREDIG's web interface.

Organism	Substrate	*k*_cat_ [1/s]	$K_{m}$ [mM]	experimental conditions	source
*Trichoderma reesei*	4-nitrophenyl lactoside	0.19	0.04	pH=5, 30Â°C	BRENDA

*Thermobifida fusca*	2,4-dinitrophenyl cellobiose	0.057	0.0023	pH=5.5, 50Â°C	BRENDA

*Rasamsonia emersonii*	2-chloro-4-nitrophenyl-beta-D-cellotrioside	8.9	4.5	pH=5, 50Â°C	BRENDA

**Table A.4:** *β*-glucosidase (EC 3.2.1.21) values tabulated on PREDIG's web interface.

Organism	Substrate	*k*_cat_ [1/s]	$K_{m}$ [mM]	experimental conditions	source
*Carapichea ipecacuanha*	ipecoside	51.6	0.1	pH=5.0, 30Â°C	BRENDA

*Thermoascus aurantiacus*	cellobiose	284	0.637	pH=5, 50Â°C	BRENDA

*Humicola insolens*	cellobiose	93.7	8.44	pH=5.5, 60Â°C	BRENDA

**Table A.5:** Xylanase (EC 3.2.1.8) values tabulated on PREDIG's web interface.

Organism	Substrate	*k*_cat_ [1/s]	$K_{m}$ [mM]	experimental conditions	source
*Trichoderma reesei*	xylohexaose	68	0.073	pH=5.0 and 20Â°C	BRENDA

*Rasamsonia emersonii*	4-nitrophenyl xylopyranoside	898	32.9	pH=5, 50Â°C	BRENDA

*Thermoascus aurantiacus*	4-methylum-belliferyl-xylotriose	833	0.067	pH=5.0, 50Â°C	BRENDA

## Appendix A $k_{\mathrm{cat}}$ and $K_{m}$ values from BRENDA

The following tables display the enzyme kinetic rates ($k_{\mathrm{cat}}$ and $K_{m}$), the source micro-organisms, the specific substrate, and the experimental conditions taken from the BRENDA database, for the cellulase and xylanase enzyme choices that are available on the web interface.

## Appendix B Parameters for running the stochastic biophysical model locally

**Parameters listed in the input file ‘simulation_parameters.txt’:**

On the web interface, these parameters are fixed and cannot be varied.

• **Maximum Gillespie steps**
  parameter numbers: 1, 2.
  It represents the maximum number of Gillespie steps. These parameters sum up to yield the maximum number of steps. Two parameters were kept in case there would exist some kind of steady state. These are fixed at a very high value for the web interface.
• **Maximum number of snapshots**
  parameter number: 3
  The parameter gives the maximal number of snapshots that may be taken for the purpose of creating animation data. It has been fixed to a high value in the web interface.
• **Maximum real time**
  parameter number: 4
  This parameter determines the maximum real time, as calculated by the Gillespie algorithm (in hours) for which the simulation will run. This has been fixed in the web interface: to a very high value (1000000) in the SIMULATION tab, and to match the highest time point for experimental data in the FITTING tab.
• **Snapshot interval**
  parameter number: 5
  This parameter determines the interval between system snapshots for recording data for rendering an animation. This number has been fixed in the web interface to 32 to optimize the viewing experience with neither too long a video nor too many missing frames
• **Maximum number of hemicellulose/lignin layers around the cellulose core** parameter number: 6
  This parameter represents the maximum number of hemicellulose/lignin layers around the cellulose core. This has been fixed to value 4 in the web interface.
• **Mode hemicellulose**
  parameter number: 7
  This parameter determines the number of sides of the microfibril, where hemicellulose can anchor. This has been fixed to 2 in the web interface.
• **Mode lignin**
  parameter number: 8
  This parameter determines on which sides of the microfibril, where lignin can anchor. This has been fixed to 2 in the web interface.
• **Mode inhibition**
  parameter number: 9
  This parameter determines whether end-product inhibition is active (1) or inactive (-1). This has been fixed in the web interface to 1.
• **Mode lignin glue**
  parameter number: 10
  This parameter determines whether the non-productive adsorption of enzymes onto lignin is active (1) or inactive (-1). This has been fixed to value 1, in the web interface.
• **Mode enzyme size**
  parameter number: 11
  This determines whether the steric hindrance due to enzyme size is included for all enzymes (1) or only for CBH (-1). This is fixed to 1 in the interface.
• **Number of Runs**
  parameter number: 12
  This determines the number of Gillespie simulation runs. This is fixed to 1 in the web interface.
• **Mean lignin covering**
  parameter number: 13
  This parameter determines the average fraction of lignin polymers which act as a structural barrier. This has been fixed to 0.5 in the web interface.
• **Sigma lignin covering**
  parameter number: 14
  This parameter represents the standard deviation around Mean lignin covering. This has been fixed to 0.2 in the web interface.

**Parameters listed in the input file ‘kinetic_parameters.txt’:**

Most of these parameters can be varied via the web interface.

• **EG**$K_{cat}$
  parameter number: 1
  Endoglucanase catalytic constant. This parameter represents the number of molecules of substrate transformed per unit of time. Units: 1/s. Range: 0.001-1000. This parameter is available to be varied on the web interface, and can also be chosen from the values in Table A.2; and can be fitted via the fitting algorithm.
• **EG**$K_{m}$
  parameter number: 2
  Endoglucanase Michaelis-Menten constant. This parameter represents the substrate concentration where the reaction velocity is half of the maximum reaction velocity under substrate saturation. Units millimolar. Range: 0.001-1000. This parameter is available to be varied on the web interface, and can also be chosen from the values in Table A.2; and can be fitted via the fitting algorithm.
• **CBH processive digestion rate**
  parameter number: 3
  This parameter represents the reaction rate of the cellobiohydrolase processive action in reactions/hour. Range: 10-10000. This parameter is available to be varied on the web interface and can also be fitted via the fitting algorithm.
• **BGL**$K_{cat}$
  parameter number: 4
  *β*-glucosidase catalytic constant. This parameter represents the number of molecules of substrate transformed per unit of time. Units: 1/s. Range: 0.001-1000. This parameter is available to be varied on the web interface, and can also be chosen from the values in Table A.4; and can be fitted via the fitting algorithm.
• **BGL**$K_{m}$
  parameter number: 5
  *β*-glucosidase Michaelis-Menten constant. This parameter represents the substrate concentration where the reaction velocity is half of the maximum reaction velocity under substrate saturation. Units millimolar. Range: 0.001-1000. This parameter is available to be varied on the web interface, and can also be chosen from the values in Table A.4; and can be fitted via the fitting algorithm.
• **XYL**$K_{cat}$
  parameter number: 6
  Xylanase catalytic constant. This parameter represents the number of molecules of substrate transformed per unit of time. Units: 1/s. Range: 0.001-1000. This parameter is available to be varied on the web interface, and can also be chosen from the values in Table A.5; and can be fitted via the fitting algorithm.
• **XYL**$K_{m}$
  parameter number: 7
  Xylanase Michaelis-Menten constant. This parameter represents the substrate concentration where the reaction velocity is half of the maximum reaction velocity under substrate saturation. Units millimolar. Range: 0.001-1000. This parameter is available to be varied on the web interface, and can also be chosen from the values in Table A.5; and can also be fitted via the fitting algorithm.
• **Lignin adhesion parameter**
  parameter number: 8
  This parameter represents the number of monolignols which undergo non-productive adsorption on a single enzyme molecule. It is a dimensionless number. Range: 150-350. This parameter is available to be varied on the web interface and can also be fitted via the fitting algorithm.
• **CBH**$K_{cat}$
  parameter number: 9
  Cellobiohydrolase catalytic constant. This parameter represents the number of molecules of substrate transformed per unit of time. Units: 1/s. Range: 0.001-1000. This parameter is available to be varied on the web interface, and can also be chosen from the values in Table A.3; and can be fitted via the fitting algorithm.
• **CBH**$K_{m}$
  parameter number: 10
  Cellobiohydrolase Michaelis-Menten constant. This parameter represents the substrate concentration where the reaction velocity is half of the maximum reaction velocity under substrate saturation. Units millimolar. Range: 0.001-1000. This parameter is available to be varied on the web interface, and can also be chosen from the values in Table A.3 and can be fitted via the fitting algorithm.
• **inhibition binding affinity of cellobiose to EG**
  parameter number: 11
  This parameter represents the fraction of the EG molecules that would be inhibited if an infinite number of inhibitor cellobiose molecules were available. It is a dimensionless number. Range: 0-1. This parameter is available to be varied on the web interface and can also be fitted via the fitting algorithm.
• **inhibition binding affinity of cellobiose to CBH**
  parameter number: 12
  This parameter represents the fraction of the CBH molecules that would be inhibited if an infinite number of inhibitor cellobiose molecules were available. It is a dimensionless number. Range: 0-1. This parameter is available to be varied on the web interface and can also be fitted via the fitting algorithm.
• **inhibition binding affinity of glucose to EG**
  parameter number: 13
  This parameter represents the fraction of the EG molecules that would be inhibited if an infinite number of inhibitor glucose molecules were available. It is a dimensionless number. Range: 0-1. This parameter is available to be varied on the web interface and can also be fitted via the fitting algorithm.
• **inhibition binding affinity of glucose to CBH**
  parameter number: 14
  This parameter represents the fraction of the CBH molecules that would be inhibited if an infinite number of inhibitor glucose molecules were available. It is a dimensionless number. Range: 0-1. This parameter is available to be varied on the web interface and can also be fitted via the fitting algorithm.
• **inhibition binding affinity of glucose to BGL**
  parameter number: 15
  This parameter represents the fraction of the BGL molecules that would be inhibited if an infinite number of inhibitor glucose molecules were available. It is a dimensionless number. Range: 0-1. This parameter is available to be varied on the web interface and can also be fitted via the fitting algorithm.
• **Digestibility ratio cellulose**
  parameter number: 16
  This parameter represents the ratio between the digestibility of crystalline and amorphous cellulose. The lower the value, the harder it is to digest crystalline bonds as compared to their amorphous counterparts. It is a dimensionless number. Range: 0.00001-0.1. This parameter is available to be varied on the web interface and can also be fitted via the fitting algorithm.
• **Digestibility ratio hemicellulose**
  parameter number: 17
  This parameter represents the ratio between the digestibility of crystalline and amorphous hemicellulose. The lower the value, the harder it is to digest crystalline bonds as compared to their amorphous counterparts. It is a dimensionless number. Range: 0.00001-0.1. This parameter is available to be varied on the web interface and can also be fitted via the fitting algorithm.
• **Enzyme Radius**
  parameter number: 18
  This parameter represents the radius of the enzyme to be considered for the steric hindrance due to excluded volume interaction. It is in units of nm. The numbers are calculated using formula 2.2 in [23], ($R_{min}={\left(3V/4\pi \right)}^{{}^{\frac{1}{3}}}=0.066⁎M^{\frac{1}{3}}$). *M* is the molecular mass of the protein in Dalton and $R_{min}$ is the minimum radius of the sphere in nm that contains the mass of the protein. The molecular masses of the enzymes have been taken from BRENDA. This parameter has been fixed to a value of 2.5 nm in the web interface.

**Parameters listed in the input file ‘initial_configuration_parameters_*.txt’:**

Most of these parameters can be varied via the web interface.

• **Mode code**
  parameter number: 1
  This parameter determines the shape of the microfibril and the number of cellulose polymers present in the microfibril. Range: 1-5. Mode code = 1 or 2, Spruce wood, 24 polymers; Mode code = 3 or 4, Mung beans, 18 polymers; Mode code = 5, Maize, 36 polymers. The user selects the shape in the web interface.
• **EG percentage**
  parameter number: 2
  This parameter represents the relative fraction of endoglucanase in the enzyme cocktail. It is a dimensionless number. Range: 0-1. This parameter is available to be varied on the web interface and can also be fitted via the fitting algorithm.
• **CBH percentage**
  parameter number: 3
  This parameter represents the relative fraction of cellobiohydrolase in the enzyme cocktail. It is a dimensionless number. Range: 0-1. This parameter is available to be varied on the web interface and can also be fitted via the fitting algorithm.
• **BGL percentage**
  parameter number: 4
  This parameter represents the relative fraction of *β*-glucosidase in the enzyme cocktail. It is a dimensionless number. Range: 0-1. This parameter is available to be varied on the web interface and can also be fitted via the fitting algorithm.
• **XYL percentage**
  parameter number: 5
  This parameter represents the relative fraction of xylanase in the enzyme cocktail. It is a dimensionless number. Range: 0-1. This parameter is available to be varied on the web interface and can also be fitted via the fitting algorithm.
• **Total number of enzyme molecules**
  parameter number: 6
  This parameter represents the total number of enzyme molecules involved in the digestion of the single simulated microfibril. It is a dimensionless integer number. This has been fixed in the web interface to a value of 50.
• **Length of microfibril**
  parameter number: 7
  This parameter represents the length of the simulated microfibril in terms of *β*1-4 bonds in the cellulose chain. It is a dimensionless integer number. This has been fixed in the web interface to a value of 200, to save on computational time due to limited resources available on the server.
• **Boolean Xyl or MLG**
  parameter number: 8
  This parameter determines whether the hemicellulose contains either xylose or mixed-linkage glucans. It is a boolean, value: 0 (MLG) or 1 (xylose). This has been fixed in the web interface to 1, as xylose is much more common in hemicellulose than mixed-linkage glucans.
• **percentage of Xylose**
  parameter number: 9
  This parameter determines the percentage of xylose in the hemicellulose. It is currently restricted to the value: 1. This has also been fixed in the web interface to 1. However, one can modify the main code to include multiple types of hemicellulose sugars, which will make this parameter useful.
• **percentage of cellulose**
  parameter number: 10
  This parameter represents the relative fraction of cellulose in the substrate composition. It is a dimensionless number. Range: 0.01-1. This parameter is available to be varied on the web interface and can also be fitted via the fitting algorithm.
• **percentage of hemicellulose**
  parameter number: 11
  This parameter represents the relative fraction of hemicellulose in the substrate composition. It is a dimensionless number. Range: 0.01-0.99. This parameter is available to be varied on the web interface and can also be fitted via the fitting algorithm.
• **percentage of lignin**
  parameter number: 12
  This parameter represents the relative fraction of lignin in the substrate composition. It is a dimensionless number. Range: 0.01-0.99. This parameter is available to be varied on the web interface and can also be fitted via the fitting algorithm.
• **percentage of acetylated hemi**
  parameter number: 13
  This parameter represents the percentage of acetylated hemicellulose out of the total hemicellulose in the microfibril. Currently restricted to a value 0, as we don't include hemicellulose acetylation in the model. This parameter is fixed to 0 on the web interface.
• **percentage of crystalline cellulose**
  parameter number: 14
  This parameter represents the fraction of crystalline bonds out of the total number of cellulose bonds. It is a dimensionless number. Range: 0-1. This parameter is available to be varied on the web interface and can also be fitted via the fitting algorithm.
• **percentage of crystalline hemicellulose**
  parameter number: 15
  This parameter represents the fraction of crystalline bonds out of the total number of hemicellulose bonds. It is a dimensionless number. Range: 0-1. This parameter is available to be varied on the web interface and can also be fitted via the fitting algorithm.
• **Fraction of defects**
  parameter number: 16
  This parameter represents the fraction of the total amorphous cellulose contained in the defects within the crystalline part. These defects arise due to various pre-treatments the substrate is subjected to before enzymatic hydrolysis. It is a dimensionless number. Range: 0-0.5. This parameter is available to be varied on the web interface and can also be fitted via the fitting algorithm.
• **Fraction of defective outer polymers**
  parameter number: 17
  This parameter represents the fraction of the outer cellulose polymers which possess defects. It is a dimensionless number. Range: 0-1. This parameter is available to be varied on the web interface and can also be fitted via the fitting algorithm.
• **Radius of monomer**
  parameter number: 18
  This parameter represents the radius of a single monomer of cellulose and hemicellulose. It is in units of nm. This is fixed in the web interface to a value of 0.6.

## Appendix C Parameters for running the fitting algorithm locally

**Parameters listed in the input file ‘fit_settings.txt’:**

On the web interface, these parameters are fixed and cannot be varied, to ensure reasonable computing times.

• the number of families $\left(N_{family}\right)$ (currently to be kept at a value of 1)
• the number of generations per family $(N_{gens}$ $\left[G_{j},\;j=1,2,..,N_{gens}]\right)$ (choose an integer greater than or equal to 1)
• the number of sub-generations per generation $(N_{subsets}$ $\left[SG_{k},\;k=1,2,...,N_{subsets}]\right)$ (choose an integer greater than or equal to 1)
• the percentage around which to vary the parameters (Δ) (choose a number between 0 and 1)
• the number of CPU threads to be used for parallel running of the simulation code $\left(N_{cores}\right)$
